# Two of a Kind? Mapping the Psychopathological Space between Obesity with and without Binge Eating Disorder

**DOI:** 10.3390/nu13113813

**Published:** 2021-10-26

**Authors:** Laura Marie Sommer, Georg Halbeisen, Yesim Erim, Georgios Paslakis

**Affiliations:** 1 Department of Psychosomatic Medicine and Psychotherapy, University Hospital of Erlangen, Friedrich-Alexander-University Erlangen-Nürnberg (FAU), 91054 Erlangen, Germany; laura.marie.sommer@outlook.de (L.M.S.); yesim.erim@uk-erlangen.de (Y.E.); 2 University Clinic for Psychosomatic Medicine and Psychotherapy, Medical Faculty, Campus East-Westfalia, Ruhr-University Bochum, Virchowstr. 65, 32312 Luebbecke, Germany; georg.halbeisen@rub.de

**Keywords:** binge eating disorder, obesity, food addiction, impulsivity, emotional eating, childhood trauma questionnaire, psychotherapy

## Abstract

(1) Background: Obesity (OB) is a frequent co-morbidity in Binge Eating Disorder (BED), suggesting that both conditions share phenotypical features along a spectrum of eating-related behaviors. However, the evidence is inconsistent. This study aimed to comprehensively compare OB-BED patients against OB individuals without BED and healthy, normal-weight controls in general psychopathological features, eating-related phenotypes, and early life experiences. (2) Methods: OB-BED patients (*n* = 37), OB individuals (*n* = 50), and controls (*n* = 44) completed a battery of standardized questionnaires. Responses were analyzed using univariate comparisons and dimensionality reduction techniques (linear discriminant analysis, LDA). (3) Results: OB-BED patients showed the highest scores across assessments (e.g., depression, emotional and stress eating, food cravings, food addiction). OB-BED patients did not differ from OB individuals in terms of childhood traumatization or attachment styles. The LDA revealed a two-dimensional solution that distinguished controls from OB and OB-BED in terms of increasing problematic eating behaviors and attitudes, depression, and childhood adversities, as well as OB-BED from OB groups in terms of emotional eating tendencies and self-regulation impairments. (4) Conclusions: Findings support the idea of a shared spectrum of eating-related disorders but also highlight important distinctions relevant to identifying and treating BED in obese patients.

## 1. Introduction

Binge Eating Disorder (BED) is the most common eating disorder (ED) in western countries, with lifetime prevalence averaging 1.57% [1]. In recognition of this growing prevalence, BED was included in the 5th edition of the Diagnostic and Statistical Manual of Mental Disorders (DSM-5) [2]. According to DSM-5 criteria, BED patients suffer from reoccurring binge eating episodes of ingesting large amounts of food in a short period of time, with associated loss of control over their food intake. Negative feelings such as shame or guilt accompany these episodes. The severity of the disorder depends on the frequency of binge eating episodes per week, with thresholds demarcating mild, moderate, severe, and extreme BED.

Obesity is a frequent co-morbidity in BED due to the high volume of food intake and lack of compensatory behaviors in BED patients [3]. In the German general population, 25.9% of men and 24.4% of women are considered obese by a body mass index (BMI) > 30 kg/m^2^ [4]. Among individuals with BED symptoms, however, 41.7% are obese, compared with only 15.8% of individuals with no history of an ED [5]. Comorbid obesity in BED patients is a major cause for concern, as obesity is associated with a wide range of physical afflictions, including cardiovascular diseases, type II diabetes, and several cancers [6], as well as with increased psychological burden, such as depression, low self-esteem, body image disturbances, perceived stress, and lowered quality of life [7]. 

The frequent co-morbidity of BED and obesity may suggest that both conditions are part of a broad spectrum of eating-related behaviors, implying that patients could transition from one condition to another [8]. This idea is consistent with recent studies linking obesity with comorbid BED and obesity without BED to shared phenotypical, in part clinical, features. For example, early life adversities such as trauma have been associated with the development of both BED and obesity [9,10], possibly due to lasting impairments in coping mechanisms and metabolic alterations due to stress [11,12]. In a similar vein, insecure attachment styles that develop in early childhood have been associated with emotional eating, eating unhealthy food, and binge eating [13], which studies link to the pathogenesis of BED and obesity [14,15]. Neurobiological studies also show that dopaminergic and glutamatergic pathways play a crucial role in developing and maintaining both conditions [16], further corroborating the idea of a shared continuum between obesity with comorbid BED and obesity without BED.

Patients with BED and comorbid obesity also differ from obese individuals without BED regarding a variety of phenotypical features. Patients with BED and obesity display higher levels of impulsivity than obese individuals without BED, in general, and especially toward food cues [17,18]. Neurobiological studies have shown that BED patients with obesity show lower activity levels in brain areas responsible for control and self-regulatory processes than obese individuals without BED [17]. Patients with BED have lower response inhibition abilities when presented with food cues in a go-no-go task, a finding associated with decreased activation of the prefrontal control network, which is active during successful no-go (withhold) trials in non-BED obese individuals [19]. Evidence also suggests a more compromised hormonal regulation of hunger and satiety in BED compared to obesity, for example, as mirrored in findings of blunted postprandial ghrelin suppression in BED compared with obesity [20]. In addition, Schulz and Laessle [21] found that depression weakens self-regulation in obese BED patients but not in obese individuals without BED, suggesting a possible link between depression and binge eating behavior. Finally, a recent study from our group showned that negative mood was associated with decreased food avoidance in obese BED patients only, but not in obese individuals without BED [22].

The evidence is less consistent on commonalities and disparities between obese patients with comorbid BED and obese individuals without BED concerning eating-related symptomatology other than binge eating. Strong concerns about shape and weight are core features BED shares with other EDs like anorexia nervosa or bulimia nervosa [23,24], and significant concerns of shape and weight also emerge in connection with obesity without BED in large community samples [25]. Similarly, several eating styles have been associated with obesity and comorbid BED in comparison with obesity without BED, such as emotional eating [22,26,27,28,29,30], eating in response to stress [31], and lower success in dieting and restraint [32], although especially research on the role of restraint has generated inhomogeneous results [33,34]. Newer concepts such as food craving and the strong desire to eat certain foods show stronger correlations with binge eating than obesity [35,36,37]. Food addiction, which among others, describes a loss of control over eating, cravings, and continued excessive food consumption contrary to the knowledge of adverse consequences [38], is also associated with a higher frequency of binge episodes and emotional eating [39,40]. At the same time, food addiction may play a role in obesity, too [41].

Further exploration of the commonalities and disparities between obesity with comorbid BED and obesity without BED is warranted, considering the results may help identify specific targets of prevention and intervention. Existing therapeutic approaches and weight loss strategies for obesity, such as dietary programs or physical exercise, are often unsuccessful or do not lead to enduring weight reductions [7,42]. Obese patients with BED also exhibit smaller weight reductions compared with obese individuals without an ED following weight-loss surgery [43,44]. Cognitive behavioral therapy (CBT), which functions as the foundation of BED treatment, aims to modify eating behaviors and has been found to lead to a remission of binge episodes among 64.4% of patients, including positive effects in terms of co-occurring psychological impairments [32,45]. However, the evidence for long-term results after cessation of CBT is still disappointing [32,46], suggesting BED treatment needs further improvement. 

Thus far, the commonalities and differences in phenotypical features between obese BED patients and obese individuals without BED have been mapped across a range of studies but are seldom explored within a single investigation. One cannot exclude that differences in study design or sample composition account for some inconsistencies across findings, suggesting the need for a more comprehensive investigation. A comprehensive investigation also allows for the assessment the relative contribution of different sets of phenotypical and clinical features in grouping and distinguishing obese patients with BED from obese individuals without BED, aiding in theory development and prognostic application. Here, we conducted an exploratory study in which we assessed eating-related symptomatology (shape and weight concerns [23,24], emotional eating [26,27,28,29,30,47], dieting [32], food craving [37], and food addiction [48]), general psychopathology (impulse control impairments [17], depression [34]), and early life experiences (childhood traumatic events [9,10], attachment styles [14,15]) using standardized questionnaires. Specifically, we aimed to comprehensively compare obese patients with BED against obese individuals without BED as well as healthy, normal-weight controls in terms of these features, using univariate analyses and dimensionality reduction techniques.

## 2. Materials and Methods

### 2.1. Participants

A total of 131 German-speaking adults (90 women, 41 men, mean age = 42.7 years, age range: 21 to 82 years) participated in the study: *n* = 37 obese patients with an active BED (OB-BED; BMI > 30 kg/m^2^), *n* = 50 weight-matched obese controls (OB; BMI > 30 kg/m^2^), and *n* = 44 healthy, normal-weight controls with a BMI between 19.0 and 24.9 kg/m^2^ (CO). OB-BED patients were recruited from the psychosomatic ward and day clinic of the Department for Psychosomatic Medicine and Psychotherapy at the University Hospital of Erlangen. Patients were both newly diagnosed individuals as well as individuals who had already received eating disorder-specific treatment in the past. University students, hospital employees, and individuals considering bariatric surgery as an option for weight loss were recruited for the OB and CO groups. For the diagnosis of BED, DSM-5 criteria (e.g., recurrent episodes of binge eating, marked distress, absence of compensatory behaviors) had to be fulfilled [2], which were assessed and confirmed by a physician with longstanding experience in diagnosing and treating eating disorders, in addition to the review of pre-existing documentation and the use of a clinical questionnaire [49]. Common inclusion criteria across groups were: 18 years or older, absence of acute severe psychiatric or somatic concomitant diseases, and no acute suicidal tendencies. ED diagnoses other than BED, or other clinically relevant ED symptoms, served as exclusion criteria; these exclusion criteria were verified before study inclusion during a clinical interview by the physician in charge.

The study was carried out in accordance with the Declaration of Helsinki and was reviewed and approved by the local ethics committee of the Friedrich-Alexander-University Erlangen-Nürnberg (approval no.: 267_17B, 4 December 2017). A sample size of *N* ≥ 130 was targeted to achieve a power of 0.70 for detecting medium- or larger-sized (i.e., *f* ≥ 0.25) group differences at *p* ≤ 0.05 [50]. Participants had no previous experience with the procedure, provided informed, written, and signed consent, and were randomly sampled by convenience among local individuals and patients that were or became available during the recruitment period. Refusals to participate were not recorded; thus, information on participation rate cannot be provided. 

### 2.2. Procedure

All participants completed a battery of paper-and-pencil questionnaires at their own pace, detailed below, to measure different aspects of eating-related symptomatology, general psychopathology, and childhood adversities.

### 2.3. Assessment of Eating-Related Symptomatology

Problematic eating behaviors and attitudes, such as shape and weight concerns, were assessed using the Eating Disorder Examination—Questionnaire (EDE-Q). The EDE-Q [51] is a self-report questionnaire modeled after the Eating Disorder Examination [52]. It is composed of 22 items and assesses four subcategories: Restraint, Eating Concern, Weight Concern, and Shape Concern. Items are rated on a 6-point scale, based on how often the eating disorder characteristics occurred within the past 28 days. Mean scores are computed for each subcategory, as well as for the overall questionnaire [53].

Emotional eating tendencies were assessed using the Salzburger Emotional Eating Scale (SEES) [54]. The SEES contains twenty items, scored on a 5-point Likert scale and grouped into four subcategories for effects of emotions on eating (happiness, sadness, anger, and anxiety), each yielding a mean score. Mean scores higher than 3 suggest an increased influence of emotion on food intake, while scores below 3 suggest a decreased influence of emotion on food intake.

The Salzburger Stress Eating Scale (SSES) [31] is a ten-item questionnaire that measures general stress eating tendencies, which we included because stress can affect eating even after controlling for the effects of negative emotions [55]. Each item is scored on a 5-point Likert scale ranging from 1 = I eat much less than usual to 5 = I eat much more than usual. A mean score is calculated using all items. Mean scores higher, or lower, than 3 indicate an increased, or decreased, intake when the individual feels stressed, respectively.

As further measures of emotional eating and restraint, we included the Dutch Eating Behavior Questionnaire (DEBQ) [56]. The scale also measures external eating, the tendency to eat after being exposed to food cues. The Emotional Eating scale further splits into effects of diffuse emotions and clearly labeled emotions. The German version has 30 items that are scored on a 5-point Likert Scale [57].

As an additional measure of restraint eating, we included the Perceived Self-Regulatory Success in Dieting (PSRS) [58], a short questionnaire that can be used for distinguishing between successful and unsuccessful dieters [59]. Three brief questions are used to assess whether respondents find it easy to watch their weight, lose weight, or find it challenging to stay in shape. The items are scored on a 7-point Likert scale, with the last item being reversed coded.

Food craving was assessed with the Food Craving Questionnaire—Trait (FCQ-T) [60]. It consists of 39 items scored on a 6-point Likert scale, which in the German version [35] are separated into six subscales: Intentions/Lack of control, Reinforcement, Thoughts/Guilt, Emotions, Cues, and Hunger.

Finally, we also included the Yale Food Addiction Scale (YFAS) 2.0 [61] in order to measure addiction-like eating behavior. The YFAS 2.0 has 35 items which assess how many of the eleven symptoms of food addiction according to DSM-5 addiction criteria (amount, attempts to quit, time, reduced activities, consequences, tolerance, withdrawal, craving, failed obligations, problems, hazardous situations) are present, as well as if the eating behavior causes impairment or distress. The items are scored on a 7-point scale, with each symptom having a specific threshold score. According to the number of symptoms present, the severity of food addiction is considered to be mild (2–3), moderate (4–5), or severe (7 or more symptoms). The endorsement of impairment/distress is necessary for diagnosing addiction at all [62].

### 2.4. Assessment of General Psychopathology

The Barratt Impulsiveness Scale—Short Version (BIS-15) [63] was used to measure participants’ impulsiveness utilizing a three-factor model: non-planning impulsivity, motor impulsivity, and attentional impulsivity. The BIS-15 contains 15 items, each scored on a 4-point Likert scale with six items scored inversely. Accordingly, a sum score of all items ranges between 15 and 60.

The Beck Depression Inventory (BDI-II) [64] was additionally included as a widely used self-report inventory for measuring the severity of depression in adults. The BDI-II contains 21 items, each scored on a 4-point Likert scale, with sum scores ranging between 0 and 63.

### 2.5. Assessment of Early Life Experiences

Two questionnaires on early life experiences were also included. The short version of the Childhood Trauma Questionnaire (CTQ) [65] contains 28 items and screens for five types of childhood trauma, including physical, sexual, and emotional abuse, as well as physical and emotional neglect. Each subcategory contains five items, and the remaining three items comprise the Minimization/Denial validity scale, which indicates underreporting of maltreatment. All items are rated on a 5-point Likert scale, with some items scored inversely, yielding a sum score for each subcategory.

Finally, the redesigned German version of the Relationship Scales Questionnaire (RSQ) [66] was used to assess attachment style and distinguished between “Separation anxiety”, “Closeness anxiety“, “Lack of trust,” and “Wish to be independent”. Each of the 30 items are ranked on a 5-point Likert scale, yielding a mean score for each subcategory of attachment.

### 2.6. Data Aggregation and Analysis

Participant responses were aggregated according to each questionnaire’s specifications. For questionnaire total scores, univariate analyses of variance (ANOVAs) were conducted to assess differences between groups (OB-BED vs. OB vs. CO). To account for multicollinearity among questionnaire subscales, between-group differences on subscales were analyzed using one-way multivariate analysis of variance (MANOVAs). Anthropometric variables (age, BMI) were compared between groups using one-way ANOVA, and a Chi-squared test of independence (for sex ratio), with group as the independent variable. Chi-square tests were also used to analyze symptom severity in the YFAS 2.0. 

For assessing the relative contribution of different sets of features in grouping and distinguishing OB-BED patients from OB and CO individuals, a linear discriminant analysis (LDA) was conducted with participant group as the criterion variable and questionnaire scores as predictor variables. The LDA’s primary goal is to identify along how many and which dimensions (i.e., the discriminant functions) the participant groups can be distinguished from each other based on a set of predictor variables. Correlations (loadings) between predictors and discriminant functions can be used to indicate the relative value of each questionnaire to the discriminant function. Prior probabilities were adjusted to control for unequal group sizes. Box’s *M* statistic was used to test for violations of the assumption of equal covariance matrices.

**Figure 1:**
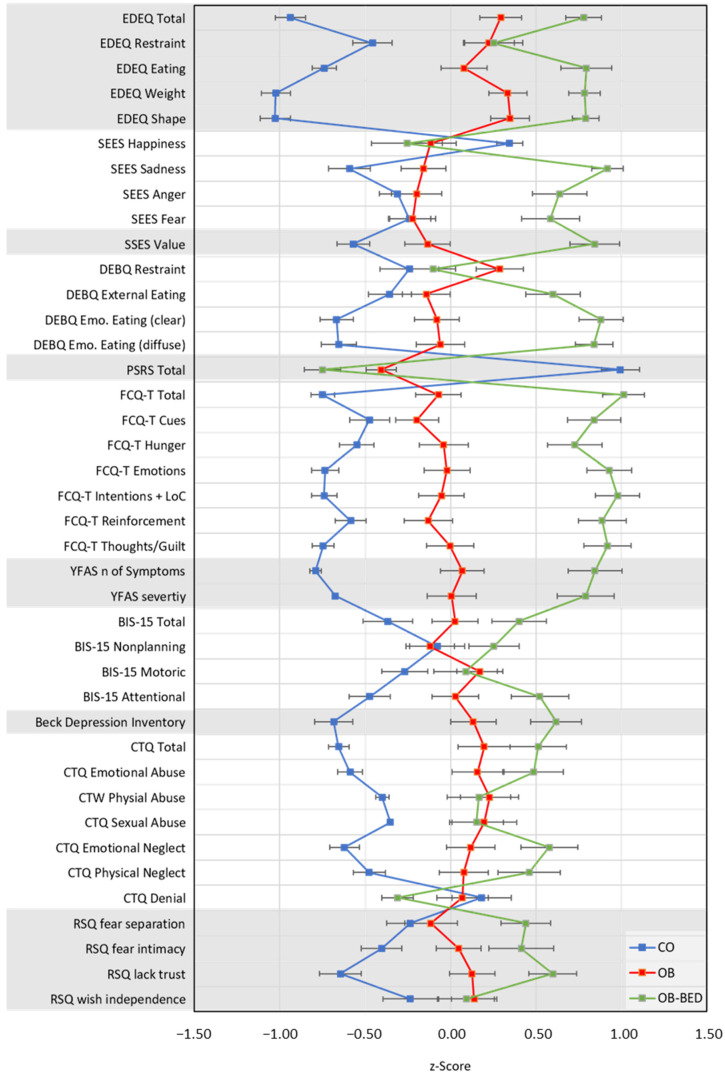
Mean questionnaire scores (z-standardized) and standard errors for the examined groups. CO = control; OB = obese; OB-BED = obese with co-morbid Binge Eating Disorder.

The significance level for all analyses was set at *p* ≤ 0.05. Effect sizes are reported as *η*^2^. Post hoc pairwise comparisons report Bonferroni-adjusted *p*-values for multiple comparisons. Variable values are reported as mean ± standard deviation. Z-standardized values of questionnaires are depicted in Figure 1. Unstandardized means ± standard deviation for questionnaire responses are summarized in Appendix A (Table A1). All data were analyzed with the Statistical Package for the Social Sciences (SPSS 25; IBM Corp., Armonk, NY, USA).

## 3. Results

### 3.1. Participant Demographics

Participant demographics are summarized in Table 1. Participant sexes were similarly distributed across participant groups, χ^2^ (df = 2) = 0.49, *p* = 0.78. Groups differed as intended in terms of BMI (post hoc: OB-BED = OB > CO), *F*(2, 128) = 125.05, *p* < 0.001, *η*^2^ = 0.66, and were similar in age, *F*(2, 128) = 3.02, *p* > 0.05.

### 3.2. Eating-Related Symptomatology: EDEQ, SSES, SEES, DEBQ, PSRS, FCTQ, & YFAS 2.0

EDE-Q total scores of disordered eating varied significantly between groups, *F*(2, 125) = 62.82, *p* < 0.001, *η*^2^ = 0.50, with OB-BED scoring higher than OB and CO, *p*s < 0.008, and OB scoring higher than CO, *p* < 0.001. Expectedly, the analysis of subcategory scores replicated the effect, *F*(8, 244) = 20.88, *p* < 0.001, *η*^2^ = 0.27, Wilk’s Λ = 0.41, with OB-BED and OB showing higher scores than CO on Restraint, Eating Concern, Weight Concern, and Shape Concern scales, *p*s < 0.002. With the exception of restraint, *p* = 1.00, OB-BED also scored consistently higher than OB, *p*s < 0.007.

Emotional eating according to SEES subscales varied by group, *F*(8, 250) = 9.23, *p* < 0.001, *η*^2^ = 0.23, Wilk’s Λ = 0.60, with OB-BED scoring lower on the happiness subcategory than CO, *p* = 0.02, but not OB, *p* = 1.00. OB-BED scored higher on the sadness, anger, and fear subcategories than both OB and CO, *p*s < 0.001. With the exception of higher sadness scores for OB than CO, *p* = 0.03, OB and CO were not significantly different, *p*s > 0.07.

SSES mean scores for general stress eating tendencies also differed significantly between groups, *F*(2, 127) = 29.77, *p* < 0.001, *η*^2^ = 0.32, with CO scoring lower than OB, *p* = 0.04, and OB-BED, *p* < 0.001, and OB scoring lower than OB-BED, *p* < 0.001.

On the DEBQ, scores differed between groups across restraint, external eating and emotional eating subscales, *F*(8, 246) = 11.31, *p* < 0.001, *η*^2^ = 0.27, Wilk’s Λ = 0.53. CO scored significantly lower on restraint eating behaviors than OB, *p* = 0.03, but not lower than OB-BED, *p* = 1.00, with OB and OB-BED remaining comparable, *p* = 0.21. On the external eating subscale, OB-BED scored higher than OB and CO, *p*s < 0.001, with OB and CO remaining comparable, *p* = 0.82. Finally, on both subscales of emotional eating, OB-BED scored higher than OB and CO, *p*s < 0.001, and OB scored higher than CO, *p*s < 0.002.

In terms of the PSRS perceived success in dieting scores, group differences were obtained, *F*(2, 120) = 80.71, *p* < 0.001, *η*^2^ = 0.57, due to CO scoring significantly higher than both OB and OB-BED groups, *p*s <.001. OB and OB-BED did not differ in terms of PSRS, *p* = 0.07.

FCTQ food craving total scores differed significantly between groups, *F*(2, 121) = 58.02, *p* < 0.001, *η*^2^ = 0.49, with OB-BED scoring higher than OB and CO, *p*s < 0.001, and OB scoring higher than CO, *p* < 0.001. This pattern reproduced consistently across FCTQ subscales (OB-BED > OB > CO, *p*s < 0.001), *F*(12, 232) = 9.10, *p* < 0.001, *η*^2^ = 0.32, Wilk’s Λ = 0.46, with the exception of the food-cue elicited craving, which did not differ between OB and CO, *p* = 0.38.

Finally, food addiction symptoms based on the YFAS 2.0 differed significantly across groups, *F*(2, 128) = 45.66, *p* < 0.001, *η*^2^ = 0.42, with OB-BED scoring higher than OB and CO, *p*s < 0.001, and OB scoring higher than CO, *p* < 0.001. A Chi-squared test of independence revealed that groups also differed in terms of the clinical significance of food addiction symptoms, χ^2^ (df = 2) = 49.97, *p* < 0.001, with 0% of CO, 40% of OB and 76% of OB-BED indicating clinically significant impairments. However, of those classified as severe food addicted (*n* = 31), 61% were in the OB-BED group and 39% were in the OB group, which did not significantly differ from the expected values based on sample size distribution, *p* = 0.67. 

### 3.3. General Psychopathology: BIS-15 & BDI

BIS-15 total impulsivity scores differed significantly between groups, *F*(2, 128) = 6.48, *p* < 0.002, *η*^2^ = 0.09, with OB-BED scoring higher than CO, *p* < 0.001, whereas OB-BED and OB, and OB and CO remained comparable, *p*s > 0.14. Subscale analysis, *F*(6, 252) = 4.56, *p* < 0.001, *η*^2^ = 0.10, Wilk’s Λ = 0.81, revealed that overall differences were due to OB-BED scoring higher on attentional impulsivity than OB and CO, *p*s < 0.05, and OB scoring higher than CO, *p* = 0.03. All groups remained comparable on non-planning impulsivity and motor impulsivity subscales, *p*s > 0.10.

On the BDI depression inventory, OB-BED scored higher than OB and CO, *p*s < 0.04, and OB scored higher than CO, *p* < 0.001, *F*(2, 126) = 23.78, *p* < 0.001, *η*^2^ = 0.27. 

### 3.4. Early Life Experiences: CTQ & RSQ

CTQ total scores for childhood trauma revealed significant groups differences, *F*(2, 128) = 19.67, *p* < 0.001, *η*^2^ = 0.24, with CO scoring lower than both OB-BED and OB, *p*s < 0.001, and similar scores for OB-BED and OB, *p* = 0.30. In terms of trauma subcategories, group differences were also significant, *F*(10, 248) = 4.51, *p* < 0.001, *η*^2^ = 0.15, Wilk’s Λ = 0.72, with CO scoring lower than OB-BED and OB in all categories, *p*s < 0.03, except for OB-BED in terms of sexual abuse, *p* = 0.06. OB-BED and OB did not significantly differ in any of the subcategories, *p*s > 0.05. Denial scores indicating underreporting did not differ between groups, *F*(2, 128) = 2.70, *p* = 0.07.

Attachment styles according to RSQ scores differed between groups, *F*(8, 244) = 6.32, *p* < 0.001, *η*^2^ = 0.17, Wilk’s Λ = 0.69, with differences found on separation anxiety, closeness anxiety, and lack of trust subscales. For separation anxiety, OB-BED revealed elevated scores compared to OB and CO groups, *p*s < 0.03, which themselves remained comparable, *p* = 1.00. Closeness anxiety was only elevated for OB-BED when compared to CO, *p* < 0.001, whereas all other comparisons were not significant, *p*s > 0.15. Finally, all groups differed in lack of trust, *p*s < 0.05, with OB-BED scoring higher than OB and CO, and OB scoring higher than CO. There were no significant differences on the wish to be independent subscale, *p*s > 0.25.

### 3.5. Discriminant Analysis

Given that in almost all questionnaires and subcategories, significant differences were found between OB-BED, OB and CO groups, a linear discriminant analysis (LDA) was conducted to assess the relative contribution of different sets of psychopathological features in grouping and distinguishing the groups. Given the high internal consistencies of SEES (with happiness reverse-coded), DEBQ, and RSQ subscales (excluding wish for independence, which did not differ between groups), Cronbach’s α 0.74, 0.71, and 0.66, respectively, mean total scores for these questionnaires were computed. Thus, eleven total scores for all questionnaires were entered as predictors in the LDA. Because of missing scores on at least one questionnaire, 116 cases were included in the analysis.

**Figure 2:**
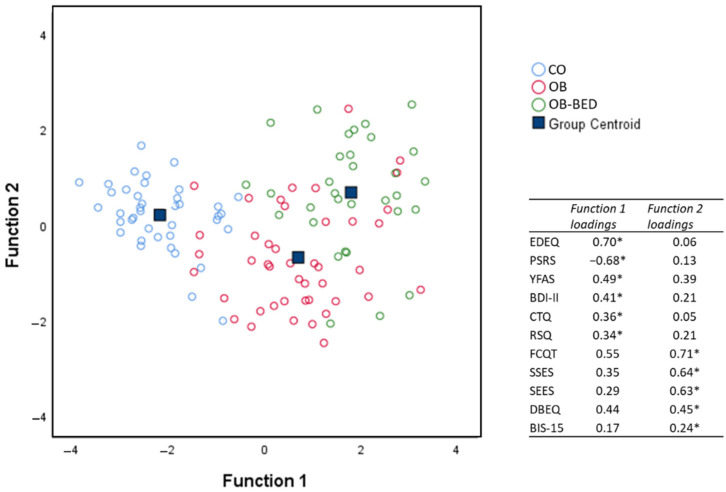
Cases and centroids of study groups on the two discriminant functions derived from the linear discriminant analysis (LDA) of questionnaire responses. Asterisks (*) indicate the largest absolute correlation between each predictor and the discriminant functions. CO = control; OB = obese; OB-BED = obese with co-morbid Binge Eating Disorder.

Results of the LDA revealed a two-dimensional solution (see Figure 2), with a significant function 1 accounting for 89.6% of the variance, χ^2^ (22) = 174.84, *p* < 0.001, and a significant function 2 accounting for the remaining 10.4% of variance, χ^2^ (10) = 30.49, *p* < 0.001. Group centroids (i.e., means in multivariate space) suggest a clear distinction between CO (−2.17), OB (0.69) and OB-BED (1.80) groups due to predictors associated with function 1, with predictors related to function 2 primarily distinguishing between OB (−0.68) and OB-BED (0.68) groups, with CO (0.21) in between. In order of importance, predictors primarily associated with function 1 (loadings in parentheses) were EDEQ (0.70), PSRS (−0.69), YFAS (0.49), BDI (0.41), CTQ (0.36), and RSQ (0.34). Function 2 predictors were FCTQ (0.71), SSES (0.64), SEES (0.63), DEBQ (0.45), and BIS-15 (0.24). Because Box’s *M* statistic indicated a significant violation of the assumption of equal covariance at *p* < 0.001, which could render LDA results unstable, a secondary LDA with only OB-BED and OB groups was conducted to confirm the reliability of function 2 predictors. Meeting assumption checks (Box’s *M p* = 0.30), the additional analysis yielded a similar solution for the distinction of OB-BED and OB groups, χ^2^ (11) = 31.64, *p* < 0.001, with FCTQ (0.87), SSES (0.74), DEBQ (0.70), and SEES (0.69) emerging again as the most important predictors of group differences. Taken together, the LDA findings suggest that CO can be distinguished from OB, and OB-BED groups along a continuum of increasing problematic eating behaviors and attitudes, depression, and childhood adversities, with OB-BED further distinguishable from OB along a continuum of increasing emotional eating tendencies, and eating-related as well as general self-regulation impairments.

## 4. Discussion

This study aimed to assess differences and commonalities between obese patients with BED and obese individuals without BED. For that, a battery of questionnaires on early life experiences, general psychopathology, and eating-related symptomatology was assessed on the above-mentioned groups and normal-weight controls, whereby the BED group was a clinically diagnosed sample. In almost all questionnaires and subcategories, significant differences could be found between the groups. However, the findings of the LDA showed that OB-BED, OB, and CO can be grouped along a two-dimensional space, with one continuum primarily distinguishing CO from the OB and OB-BED groups and a second continuum distinguishing between OB and OB-BED groups. Consistent with previous research, aspects of early life experiences emerged as shared features of OB and OB-BED groups, whereas general psychopathology in terms of impulse control impairments distinguished between the groups. However, across both dimensions, features of eating-related symptomatology emerged as the most important predictors of commonalities and disparities between obesity with and without BED. These results are discussed in further detail in the following sections.

### 4.1. Eating-Related Symptomatology

Although not included in the DSM-5 diagnostic criteria [2], strong concerns about shape and weight are core psychopathologies that BED shares with other EDs like anorexia nervosa or bulimia nervosa [23,24]. Significant concerns of shape and weight and elevated measures of eating disturbances also emerge in connection with obesity in large community samples [25]. For example, Hilbert et al. [67] reported an increased risk of 11 to 20 times for obese individuals to show eating disorder psychopathology compared with individuals with normal weight. Consistent with these findings, OB-BED patients scored consistently higher on the EDEQ than OB participants, who scored higher than controls, with the subscale restraint as the only exception (for similar findings, see [68]). The LDA also suggests that the OB and OB-BED groups can be distinguished from controls along a continuum best described by elevated eating disturbances. Although more research on the relative importance of restraint appears warranted (cf. [69]), these findings suggest that shape and weight concerns in obese BED patients should be of special interest since they may be related to the condition’s pathogenesis and can determine the therapy outcome [70]. 

Related to the question of the role of restraint, we assessed the PSRS. This short questionnaire yields the individuals’ self-assessment of their own success in dieting, which has been shown to negatively correlate with BMI, rigid dietary control, food cravings, food addiction symptoms, and binge eating, but to correlate positively with flexible dietary control [59]. In our study, the OB-BED and OB groups reported much lower scores in dieting successfully than controls but remained comparable, which was also indicated by the LDA associating the PSRS with the continuum relevant to the distinction of healthy controls from OB and OB-BED groups. A possible explanation for the lack of distinction between OB-BED and OB groups according to restraint is that the questionnaire measures the attempt to lose weight, rather than actual restraint eating behavior, which more specifically relates to disordered eating [71,72].

However, OB-BED and OB groups were clearly distinguishable according to features of emotional eating, as suggested by the LDA and individual analyses. In the SEES, which was developed with the expectation that persons with lower eating pathologies tend to eat rather more when happy, and persons with higher eating pathologies more when having negative emotions [54]; the average score of eating under negative emotions was increased in OB-BED compared with both OB and controls. Controls, instead, reported eating more when feeling happy. The differences between OB-BED and OB were most pronounced for sadness as a low arousal emotion, which is consistent with similar findings for binge eating in patients with bulimia nervosa [73]. These findings may suggest that mechanisms of decreased food uptake while experiencing high arousal in the form of emotions are decoupled in BED with comorbid obesity but not in obesity without BED. This idea is further supported by similar patterns found for stress eating tendencies in the SSES. Again, the OB-BED group showed the highest score by far, while the OB group reported to eat only slightly more. Controls instead reported eating less under stress. These findings are consistent with laboratory studies showing an increased speed of food uptake after stress exposure in BED patients [74].

In line with the above-summarized findings, the DEBQ, which also measures emotional eating on one subscale, was found to distinguish between OB-BED and OB groups. The OB-BED group in our study showed the highest scores in emotional eating in the DEBQ, including the subscales for diffuse emotions [56]. It stands to reason that these differences hint at the role of emotional dysregulation in predicting binge eating behavior [75].

Further lines of distinction between OB-BED and OB groups emerged along with external eating and food craving tendencies. Consistent with previous findings [75,76,77], OB-BED patients scored higher than both OB and controls on the DEBQ external eating subscale, which measures the tendency to eat after being exposed to food cues. In a similar vein, OB-BED, OB, and controls were found to differ consistently across FCQT’s food craving subscales. Although strong food cravings can be found in healthy individuals, too, it has been shown that those with binge eating symptoms score higher in food craving assessments [37]. Consistent with our findings, this association is stronger for binge eating than for only obesity [35,36,37]. Innamorati et al. [78] even developed a potential cut-off score (157.5) of the FCQT for identifying clinical-level binge eating. Although our clinically diagnosed BED sample did not meet this criterion on average (142.3), findings from the LDA, which identified FCQT scores among the most important predictors for distinguishing between OB-BED and OB groups, generally support this contention.

As BED has been linked to high impulsivity and related conditions like substance use disorders [32,79], it has been suggested that BED might share features with food addiction [41,48]. Extending this line of research, we compared OB-BED, OB, and controls in terms of food addiction as measured by the YFAS 2.0. In our study, the highest prevalence of 76% for food addiction was found in the OB-BED group, followed by OB (40%) and controls (0%). For the OB-BED group, this is a lower prevalence than shown in previous studies [38,80], though it should be mentioned that food addiction prevalence rates are inhomogeneous across different samples [41]. Given the clear difference in food addiction prevalence between OB-BED and OB groups, it might be considered surprising to find that food addiction in the LDA was associated with the more general distinction between healthy and clinical groups, rather than between OB-BED and OB groups. It should be noted, however, that the LDA included scores for food addiction symptoms rather than scores for food addiction severity, rendering these findings only partly comparable. The decision to include food addiction symptoms rather than severity scores in the LDA was mainly due to statistical reasons, as the absence of variance in severity scores of controls (i.e., none of the controls were classified as food addicted) violated the LDAs assumptions.

### 4.2. General Psychopathology

Looking at general psychopathology associated with BED, we investigated the role of impulse control impairments in relation to comorbid obesity using the BIS-15. Impulsivity has been frequently investigated in BED in the past. Experimental studies revealed that patients with BED show higher rash-spontaneous behavior, especially toward food, but also in general [17]. Neurobiological findings also link BED to impulsive/compulsive disorders, based on findings of the corticostriatal circuitry regulation of motivation and impulse control [81]. Likewise, the link between impulsivity and obesity has been of interest. A study gathering data about impulsivity in a large sample of the general population showed an association with obesity [82]. However, research suggests that BED surpasses obesity without BED in terms of impulsivity [17], which is consistent with the present findings. Specifically, OB-BED patients scored overall higher than OB and control participants on the BIS-15, with OB participants exceeding controls only on the subscale of attentional impulsivity. This scoring is in line with the findings of Loeber et al. [83], and further supported by the LDA associating impulsivity scores more strongly with the specific distinction between OB-BED and OB groups.

Further replicating previous findings on the relation between depressive symptoms and BED and obesity [84,85], we found that BDI scores of OB-BED patients exceeded those of OB, which exceeded those of controls. Interestingly, and although previous studies associated high BDI scores and depressed moods specifically with BED [21,22], the LDA suggests a linear transition from healthy controls to OB and OB-BED groups with increasing levels of depression. However, the effects of depression on BED are likely mediated through their effect on self-regulation impairments [21,86], which our analysis also suggested are more specific for the distinction of obese patients with and without obesity.

### 4.3. Early Life Experiences

Finally, adverse childhood events have been repeatedly linked to both obesity and BED [9,10]. It has been argued that adverse experiences may impair coping mechanisms due to mental and emotional perturbations but may also link to metabolic alterations due to stress, and in doing so, promote the development of obesity and BED [11,12]. Consistent with these findings, individual analysis as well as LDA groupings suggest that OB and OB-BED groups can be distinguished from healthy, normal-weight controls on the basis of overall CTQ trauma scores. However, whereas previous research indicated further distinctions between OB-BED and OB groups in terms of traumatic experiences [11,87] these differences were not significant in the present sample. In part, this may have been caused by a smaller sample size of our study or because the sexual and physical abuse scores were slightly higher in the OB group than in the OB-BED group (for similar findings, see [88]). Though further research on more specific contributions may be required, our findings corroborate childhood trauma as a general risk factor for obesity and BED, which should be considered whilst offering treatment.

In a similar vein, attachment styles, formed in early childhood, have been considered in the pathogenesis of BED and obesity [14,15] and emerged as predicting differences of OB-BED and OB groups towards controls. Specifically, OB-BED patients showed elevated RSQ scores for separation anxiety, closeness anxiety, and lack of trust, whereas OB participants differed from controls only with respect to lack of trust. To our knowledge, this is the first study assessing the RSQ in comparing groups of BED, obesity, and normal-weight controls. The findings could suggest that binge eating in obese individuals might result from poor emotional coping mechanisms [89,90,91]. However, both individual analysis and the LDA did not identify differences in attachment styles as specifically differentiating between OB-BED and OB groups, suggesting that further research on the role of attachment styles in BED is needed. 

### 4.4. Limitations

Of course, interpreting these findings is subject to limitations. First, the current study investigated the psychopathological space between obese individuals with and without BED in a cross-sectional design. Although the evidence largely supports the idea of BED and obesity forming part of a broad spectrum of eating-related behaviors and disorders in terms of childhood adversities and different aspects of general and eating-related psychopathology, the cross-sectional design prevents any exploration of a transition of individuals from one group to another. This gap may be addressed in studies using similar methods but with a longitudinal design. Second, due to the fact that we included both incident (treatment-naive) as well as prevalent (treatment-experienced) patients, we cannot exclude the possibility that prior recommendations and treated aspects of eating disorder-specific pathology could have modified the answers collected in the questionnaires, limiting interpretations as to whether the observed patterns are sensitive to change. Third, and relatedly, it must be noted that control participants were primarily recruited among individuals considering bariatric surgery as an option for weight loss (OB group), hospital staff, and medical students (normal-weight controls). Although participant groups were comparable in terms of sociodemographic features, we cannot exclude that the observed data pattern is sensitive to sample composition differences.

## 5. Conclusions

This study sought to show and reproduce specific characteristics of BED with comorbid obesity, especially in comparison with obesity without BED. Importantly, we only tested clinically diagnosed BED patients and obese individuals without a history of ED to make a clear comparison. The findings underline the distinct psychological and psychopathological features that separate OB-BED from obesity. Although OB-BED and OB groups share problematic eating behaviors and attitudes, depression, and adverse early life experiences, increasing emotional eating tendencies and eating-related and general self-regulation impairments appear to specifically relate to the emergence of BED. These results should be considered in therapy and when screening for BED in obese individuals.

## Figures and Tables

**Table 1:** Demographic information as a function of participant group.

Variable	CO	OB	OB-BED
*n* (m, f)	14, 30	17, 33	10, 27
age	41.7 ± 16.8	46.1 ± 10.7	39.3 ± 11.2
BMI (kg/m^2^)	22.5 ± 1.6	42.9 ± 9.1	46.3 ± 9.3

*n* = number of participants; m = male; f = female; BMI = body mass index; CO = control; OB = obese; OB-BED = obese with co-morbid Binge Eating Disorder. Values report mean ± standard deviation.

## Data Availability

The data presented in this study are available on request from the corresponding author.

## Appendix A

Unstandardized means ± standard deviations of questionnaire responses are summarized in Table A1.

**Table A1:** Questionnaire responses raw scores as a function of participant group.

Variable	CO	OB	OB-BED
**EDEQ**			
Total	0.7 ± 0.86	2.5 ± 1.26	3.2 ± 0.90
Restraint	0.8 ± 1.13	1.8 ± 1.54	1.8 ± 1.49
Eating Concern	0.2 ± 0.71	1.5 ± 1.43	2.5 ± 1.35
Weight Concern	0.7 ± 1.01	3.1 ± 1.43	3.9 ± 1.00
Shape Concern	1.1 ± 1.08	3.6 ± 1.47	4.4 ± 0.84
**SEES**			
Happiness	3.0 ± 0.28	2.7 ± 0.59	2.7 ± 0.71
Sadness	3.2 ± 0.60	3.5 ± 0.68	4.3 ± 0.42
Anger	2.9 ± 0.59	2.9 ± 0.88	3.6 ± 0.81
Fear	2.7 ± 0.70	2.7 ± 0.81	3.4 ± 0.88
**SSES**			
Total	2.8 ± 0.53	3.1 ± 0.79	3.9 ± 0.74
**DEBQ**			
Restraint	2.3 ± 0.91	2.7 ± 0.79	2.4 ± 0.65
External Eating	2.9 ± 0.61	3.0 ± 0.73	3.6 ± 0.73
Emo. Eating total	1.9 ± 0.69	2.6 ± 0.98	3.7 ± 0.74
Emo. E. Clearly Labelled	1.8 ± 0.73	2.5 ± 1.05	3.6 ± 0.90
Emo. E. Diffused	2.2 ± 0.78	2.9 ± 1.16	4.0 ± 0.78
**PSRS**			
Total	14.8 ± 3.72	7.8 ± 2.99	6.1 ± 3.07
**FCQT**			
Total	68.9 ± 18.81	97.2 ± 37.58	142.3 ± 30.03
Cues	10.2 ± 3.29	11.4 ± 3.64	15.8 ± 3.92
Hunger	8.8 ± 2.92	11.1 ± 4.29	14.4 ± 4.10
Emotions	6.5 ± 3.00	10.6 ± 5.24	16.1 ± 4.47
Intentions/Lack of Control	15.1 ± 5.41	22.8 ± 10.04	34.3 ± 8.57
Reinforcement	15.0 ± 5.43	19.2 ± 8.88	28.6 ± 7.71
Thoughts/Guilt	13.3 ± 4.91	22.1 ± 11.17	33.1 ± 9.71
**YFAS**			
No. of Symptoms	0.1 ± 0.78	3.1 ± 3.15	5.8 ± 3.35
Severity, n			
None	44	32	10
Mild	0	3	3
Moderate	0	2	5
Severe	0	12	19
**BIS**			
Total	29.3 ± 6.40	31.9 ± 6.34	34.4 ± 6.42
Nonplanning	10.8 ± 3.40	10.6 ± 3.20	11.8 ± 2.84
Motoric	10.1 ± 2.44	11.3 ± 2.64	11.1 ± 3.14
Attentional	8.5 ± 2.45	10.0 ± 2.96	11.5 ± 3.13
**BDI**			
Total	6.2 ± 7.61	14.7 ± 9.67	19.7 ± 9.30
**CTQ**			
Total	31.9 ± 6.16	45.2 ± 16.76	50.2 ± 15.75
Emotional Abuse	7.2 ± 2.62	11.1 ± 5.58	12.9 ± 5.60
Physical Abuse	5.3 ± 0.78	7.1 ± 3.54	6.9 ± 3.32
Sexual Abuse	5.0 ± 0.15	7.0 ± 4.83	6.8 ± 3.47
Emotional Neglect	8.3 ± 3.02	12.2 ± 5.24	14.6 ± 5.32
Physical Neglect	6.1 ± 1.83	7.8 ± 3.03	8.9 ± 3.32
Denial	0.6 ± 0.99	0.5 ± 0.91	0.2 ± 0.48
**RSQ**			
Fear Separation	2.5 ± 0.69	2.6 ± 0.79	3.0 ± 0.65
Fear Intimacy	2.2 ± 0.65	2.5 ± 0.76	2.8 ± 0.94
Lack Trust	2.0 ± 0.74	2.7 ± 0.85	3.2 ± 0.77
Wish Independence	3.8 ± 0.75	4.1 ± 0.67	4.0 ± 0.70

CO = control; OB = obese; OB-BED = obese with co-morbid Binge Eating Disorder; EDEQ = Eating Disorder Examination—Questionnaire; SEES = Salzburger Emotional Eating Scale; SSES = Salzburger Stress Eating Scale; DEBQ = Dutch Eating Behavior Questionnaire; PSRS = Perceived Self-Regulatory Success in Dieting; FCQT = Food Craving Questionnaire—Trait; YFAS = Yale Food Addiction Scale; BIS = Barratt Impulsiveness Scale – Short Version; BDI = Beck Depression Inventory; CTQ = Childhood Trauma Questionnaire; RSQ = Relationship Scales Questionnaire. Values report mean ± standard deviation.
